# Efficacy and safety of pomalidomide and low-dose dexamethasone in Chinese patients with relapsed or refractory multiple myeloma: a multicenter, prospective, single-arm, phase 2 trial

**DOI:** 10.1186/s12885-022-09802-y

**Published:** 2022-07-01

**Authors:** Wei-Jun Fu, Ya-Fei Wang, Hong-Guo Zhao, Ting Niu, Bai-Jun Fang, Ai-Jun Liao, Hai Bai, Jin Lu

**Affiliations:** 1 Department of Hematology, Changzheng Hospital, Shanghai, China; 2 Department of Hematology, Shanghai Fourth People’s Hospital, School of Medicine, Tongji University, Shanghai, China; 3 Department of Hematology, Tianjin Medical University Cancer Institute and Hospital, Tianjin, China; 4 Department of Hematology, The Affiliated Hospital of Qingdao University, Qingdao, Shandong Province China; 5 Department of Hematology, West China Hospital, Sichuan University, Chengdu, China; 6 Department of Hematology, Affiliated Cancer Hospital of Zhengzhou University and Henan Cancer Hospital, Zhengzhou, China; 7 Department of Hematology, Shengjing Hospital of China Medical University, Shenyang, Liaoning China; 8 Department of Hematology, The 940th Hospital of Joint Logistics Support Force of Chinese People’s Liberation Army, Lanzhou, China; 9 Department of Hematology, Peking University People’s Hospital and Peking University Institute of Hematology, Beijing, China

**Keywords:** Relapsed/Refractory Multiple Myeloma, Chinese Patients, Oral, Pomalidomide, Efficacy, Safety

## Abstract

**Background:**

Pomalidomide in combination with dexamethasone has demonstrated positive results in patients with relapsed or refractory multiple myeloma (RRMM), but no data are available in China. We conducted a multicenter, single-arm trial to examine the efficacy and safety of bioequivalent generic pomalidomide plus low-dose dexamethasone in Chinese RRMM patients.

**Methods:**

Adult (≥ 18 years of age) RRMM patients who progressed after at least two previous treatments, including bortezomib and lenalidomide, were eligible. Pomalidomide was given orally at 4 mg/day on days 1 to 21 of a 28-day cycle. Dexamethasone was given at 40 mg/day (either orally or intravenously; 20 mg/day at 75 years or older) on days 1, 8, 15, and 22 of each cycle. Treatment continued until disease progression or intolerable adverse events (AEs). The primary end point was objective response rate (ORR).

**Results:**

Seventy-four patients were enrolled between February 2017 and February 2019. All patients had progressed within 60 days of their last therapy. 74.3% of the patients were resistant to lenalidomide, 31.1% had renal insufficiency and 33.8% had high-risk cytogenetic RRMM. The median follow-up duration was 33.0 months (range 31.1–34.8 months). The ORR was 37.8% in the overall analysis, 32.7% in lenalidomide-refractory patients, 36.0% in patients with high-risk cytogenetics and 34.8% in RRMM patients with renal impairment. The median progression-free survival was 5.7 months (95% CI 3.7–8.8 months). The median overall survival was 24.3 months (95% CI 14.4–41.1 months). The most common grade 3 and 4 treatment-emergent adverse events (TEAEs) were neutropenia (63.5%), leukopenia (37.8%), thrombocytopenia (28.4%), and anemia (31.1%). Pulmonary infection (27.0%) was the most frequent grade 3 and 4 nonhematologic TEAE. No previously unreported AEs were observed. No venous thromboembolism was reported.

**Conclusions:**

Pomalidomide in combination with low-dose dexamethasone is effective and safe in Chinese RRMM patients.

**Trial registration:**

The study is registered at Chinese Clinical Trial Registry (ChiCTR) (ChiCTR-OIC-17013234, first registered on 03/11/2017).

**Supplementary Information:**

The online version contains supplementary material available at 10.1186/s12885-022-09802-y.

## Background

The standardized prevalence and incidence of multiple myeloma (MM) were 5.68 (5.64–5.72) and 1.15 (1.11–1.19) per 100,000 population, respectively, in Mainland China between 2012 and 2016 [1]. Though MM is less common in Chinese than in Caucasians, its incidence is increasing over time, and it is estimated that there will be 22,450 new cases of MM and 17,360 deaths in 2022 in China [2, 3]. Bortezomib, lenalidomide, and dexamethasone (VRd) induction therapy followed by lenalidomide maintenance for standard-risk patients has long-term favorable follow-up outcomes and remains the currently internationally accepted treatment regimen. However, eventual emergence of lenalidomide resistance is a major challenge to further improving survival.

High-risk cytogenetics and renal impairment are also major challenges. MM patients with high-risk cytogenetics carry an adverse outcome as they are more likely to experience relapse because of persistence of residual disease [4]. Besides, the dose of lenalidomide needs to be adjusted in patients with renal impairment [5–7]. Pomalidomide, a thalidomide analogue and a third-generation immunomodulatory drug, does not exhibit cross resistance and its dose does not need to be adjusted according to renal function. Currently, treatment options are limited for relapsed or refractory MM (RRMM) patients in China, especially in patients resistant to lenalidomide [8].

The original pomalidomide was approved by the FDA in 2013.Pomalidomide in combination with low-dose dexamethasone has proven efficacious in 2 major studies (MM-002 and MM-003) in Caucasian patients with RRMM who had received at least 2 prior therapies including bortezomib and lenalidomide. Its efficacy in patients with renal impairment was further demonstrated in the MM-013 trial. A phase 2 trial (AMN-001) has proven that pomalidomide in combination with low-dose dexamethasone is highly effective and well tolerated in Asians with RRMM [9]. However, it has not been available in Mainland China. On the other hand, there is an urgent need for treatment with pomalidomide. So when the original pomalidomide patent expired,.ChiaTai Tian Qing developed China’s first generic pomalidomide and conducted a bioequivalence study in healthy subjects (Additional file 1), which validated the bioequivalence and led to the conditional approval of the generic pomalidomide for Chinese RRMM patients in November 2020.Due to Chinese drug registration regulation that clinical trial data from mainland China should be used to support the regulatory approval of a generic drug, we conducted the prospective, single-arm trial to investigate the efficacy and safety of the generic pomalidomide in combination with low-dose dexamethasone in Chinese patients with RRMM.

## Methods

### Patients

This study enrolled adult (≥ 18 years of age) MM patients who relapsed or had disease progression while on or within 60 days after completing their last treatment despite the receipt of at least 2 prior antimyeloma therapies, including lenalidomide and bortezomib. Patient eligibility criteria were: 1) patients who had measurable disease with at least one of the following: a) serum M protein level was ≥ 0.5 g/dL, b) 24-h urinary M protein level was ≥ 200 mg, or c) the involved free light chain (FLC) level was ≥ 10 mg/dL provided that serum FLC ratio was abnormal; 2) serum creatinine was ≤ 3.0 mg/dL or calculated creatinine clearance was ≥ 30 mL/min. Additional criteria are described in Supplementary Methods.

The study protocol adhered to the SPIRIT statement and was approved by the ethics committee of all participating institutions (Additional file 2) [10]. All study subjects provided written informed consent prior to enrollment. The trial is registered with chictr.org.cn (ChiCTR-OIC-17013234, first registered on 03/11/2017). The trial was designed and conducted in accordance with the principles of Good Clinical Practice according to the International Conference on Harmonisation requirements and the Declaration of Helsinki. The study design was decided by the sponsor in collaboration with the study steering committee. All authors and the sponsor were involved in the data gathering, analysis, review, and interpretation, and writing of the report. The corresponding author had the final responsibility for the decision to submit the paper for publication.

### Treatment schedule

Pomalidomide was given orally (4 mg once daily on days 1 to 21) plus low-dose dexamethasone (40 mg/day on days 1, 8, 15, and 22, orally) of a 28-day cycle. Dexamethasone dose was reduced to 20 mg/day in all patients older than 75 years. Treatment was discontinued upon disease progression or unacceptable toxicity. The treatment cycle was interrupted if US National Cancer Institute Common Terminology Criteria for Adverse Events (CTCAE 4.0) grade 3 or 4 toxicities emerged. Growth factor support was provided without restriction and at the discretion of the attending physician. Other medications including bisphosphonates, antibiotics, analgesics, antihistamines, red blood cells, platelets or fresh frozen plasma were allowed to manage MM or its complications. Preventative aspirin, low molecular weight heparin, heparin or warfarin was prescribed to maintain the INR at 2.0 at the discretion of the attending physicians. Patients were not allowed to accept other types of anti-tumor therapy until progressive disease (PD) was confirmed.

### Clinical assessments

Samples for routine laboratory tests such as blood routine, urine routine, liver function and kidney function, coagulation function, serum and urinary M protein were obtained at enrollment, and at each assessment. Radiological studies, bone marrow examinations including smears, fluorescence in situ hybridization (FISH), immunotyping and immunohistochemistry and 12-lead electrocardiography (ECG) were performed at enrollment and each assessment. Patients underwent efficacy assessment according to the IMWG Uniform Response criteria at the end of each treatment cycle as recommended in the 2015 China Treatment Guidelines for Multiple Myeloma by an Independent Review Advisory Committee (IRAC) and investigators [11, 12]. The objective response rate (ORR) was the primary end point, defined as the proportion of patients in the intention-to-treat (ITT) population who achieved stringent complete response (sCR), CR, very good partial response (VGPR) and PR. Secondary end points included duration of response (DOR), progression-free survival (PFS) and overall survival (OS). DOR was the interval from the first PR or better to the first day of PD or death of any cause. PFS was calculated from the day when therapy was initiated to the first day of recurrence, death of any cause or the last day of follow-up. OS was calculated from the day when therapy was initiated to the day of death of any cause or the last day of follow-up. A high-risk cytogenetic profile was defined by the detection of a del17p, t(14;16), or t(4;14) cytogenetic abnormality on FISH [13, 14].

### Safety assessment

Vital signs and toxicities were assessed weekly using CTCAE version 4.0. Safety events included AEs and serious AEs (SAEs). Safety assessments were based mainly on the occurrence, frequency, and severity of AEs. Where necessary, patients were withdrawn from the study because of disease progression, withdrawal, poor compliance, or grade 2 to 3 AEs. All patients were followed until recovery from any treatment-related AE.

### Statistical analysis

By assuming single-arm α = 5% (2-sided) and β = 20%, with a reported ORR of 12% and an estimated ORR of 25% for the pomalidomide group, the target sample size was calculated using PASS 2008 to be 59. By assuming a dropout rate of at least 20%, the target sample size was set at 70.

Statistical analyses were prespecified and followed the ITT principle and undertaken using the SAS software package, version 9.3 (SAS Institute Inc., Cary, NC). The full analysis set (FAS) included all patients who received at least one dose of pomalidomide and had a baseline assessment and at least one post-baseline efficacy assessment. The per protocol set (PPS) included patients who met the eligibility criteria, received at least one dose of pomalidomide and had a baseline assessment and at least one post-baseline efficacy assessment, and did not have major study protocol violations. The safety set included all patients who received at least one dose of the study treatment. The ORR and its 95% confidence interval (CI) were calculated. The DOR, PFS and OS were calculated using the Kaplan–Meier estimate method. All tests were two-tailed with a level of significance set at α = 0.05.

## Results

### Patient demographic and baseline characteristics

**Fig. 1:**
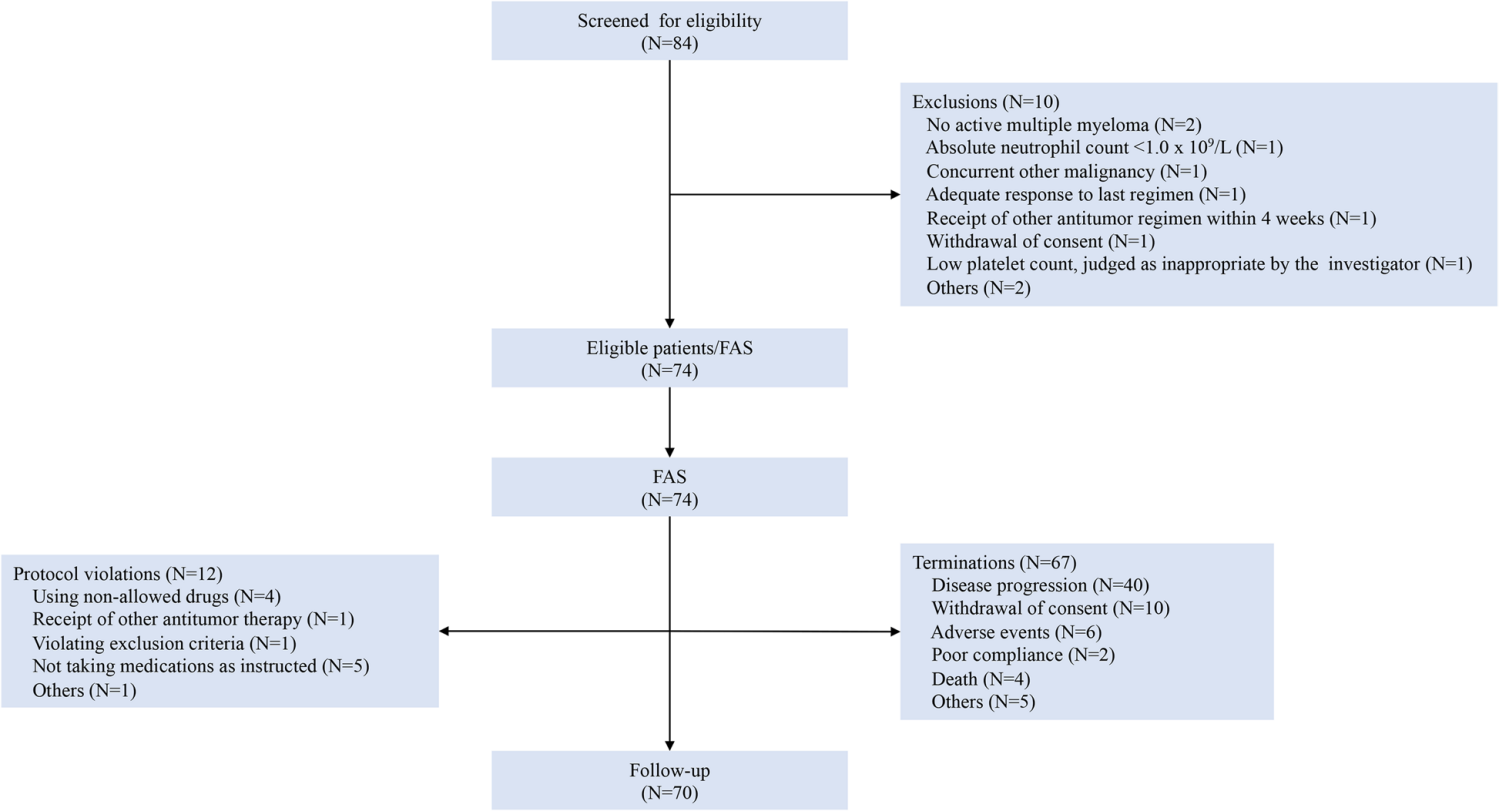
The study flowchart

The trial was conducted between February 2017 and February 2019. The flowchart is shown in Fig. 1. Eighty-four patients were screened for eligibility and 74 patients were included in the FAS. There were 44 (59.5%) males and 30 (40.5%) females, and their median age was 61 years (range 44.0–77.0 years). Patients had received a median of 3 (range1-9) prior therapies, including bortezomib and lenalidomide, and 74.3% patients were refractory to lenalidomide. Additionally, 31.1% patients had renal insufficiency, 33.8% patients had RRMM with high-risk cytogenetics, and 10.8% patients had undergone stem cell transplantation. The median number of treatment cycles was 7 (range 1–24). Patient demographic and baseline characteristics are shown in Table 1.

**Table 1:** Patient demographic and baseline characteristics

Characteristics	N(%)^a^
**Age, years**	
Median(range)	61(44–77)
≥ 65	28(37.8)
**Sex**	
Male/Female	44/30(59.5/40.5)
**Ethnicities**	
Han	71(96.0)
Others	3 (4.0)
**Median time (range) from initial diagnosis, years**	3.4(2.2–6.2)
**ECOG PS score**	
0–1	66(89.2)
2	8(10.8)
**R-ISS stage**	
I	19(25.7)
II	42(56.8)
III	13(17.6)
**Renal insufficiency** **(30 mL/min ≤ creatinine clearance < 60 mL/min), n(%)**	
Yes	23(31.1)
**Cytogenetic risk**	
Standard	49 (66.2)
High risk^b^	25(33.8)
**No. of prior lines, median(range)**	3(1–9)
**ASCT, n(%)**	
Yes	8(10.8)
**Prior MM therapy**	
Yes	74(100.0)
Bortezomib	74(100.0)
Lenalidomide	74(100.0)
**Refractory to**^c^	
Lenalidomide	55(74.3)
Bortezomib	39(52.7)
Bortezomib + Lenalidomide	32(43.2)

^a^Unless otherwise noted, all data are n(%)

ASCT: autologous stem cell transplantation

^b^High risk is defined as t(4;14), t(14;16), and/or del(17p)

Percentages calculated as percentage of risk available

^c^Refractoriness was based on most recent prior medication

### Efficacy

**Fig. 2:**
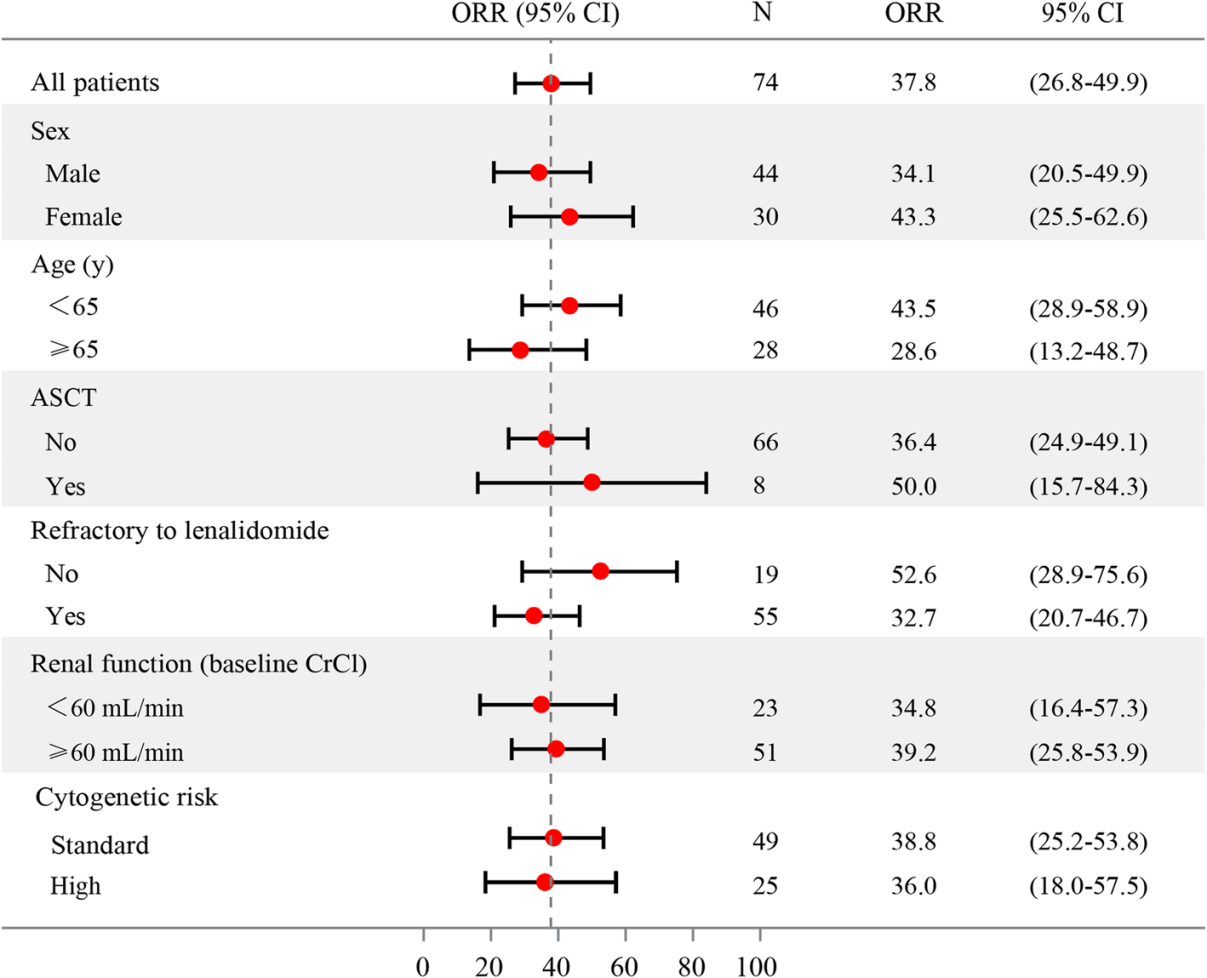
Subgroup analysis of the overall best response

**Fig. 3:**
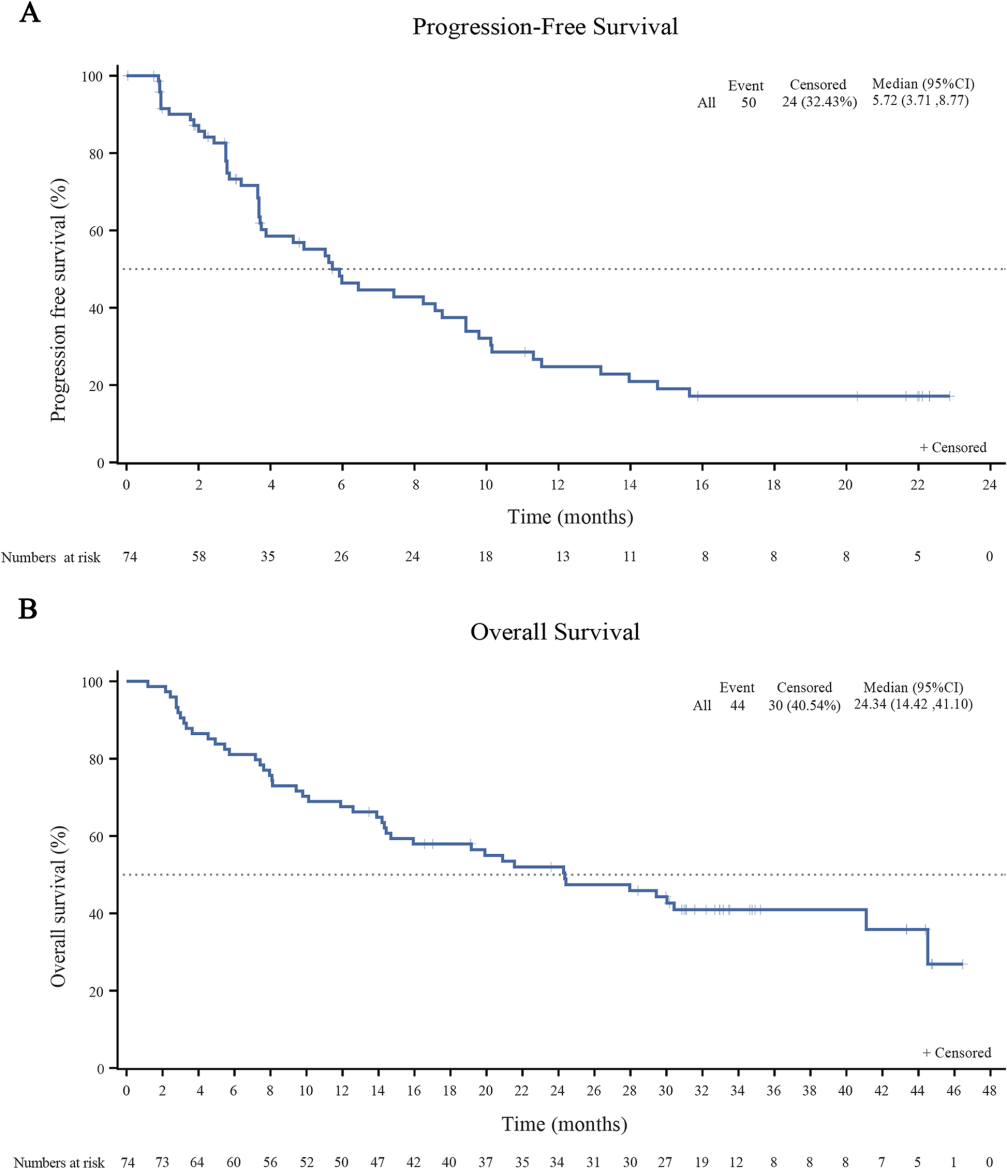
Kaplan–Meier plots in the intention-to-treat population. **a**): progression-free survival **b**): overall survival

The patients were followed up for median duration of 33.0 months (range 31.1–34.8 months). No patient was lost to follow up. The ORR, the primary end point of the study, of the FAS was 37.8% per IRAC evaluation. Three (4.1%) patients achieved CR, 5 (6.8%) and 20 (27.0%) achieved VGPR and PR, respectively. The time to response was 1.84 months (range 0.95–2.35 months) (Table 2). In addition, 32 (43.2%) patients had SD and only 5 (6.8%) patients experienced PD. Our subgroup analysis revealed that all subgroups benefited from pomalidomide and low-dose dexamethasone regimen (Fig. 2). Specifically, the ORR was 36.0% for RRMM patients with high-risk cytogenetics and 38.8% for those without. Furthermore, the ORR was 34.8% for RRMM patients with renal impairment and 39.2% for those without. The median DOR was 8.8 months (95% CI 6.0–14.7 months). The median PFS was 5.7 months (95% CI 3.7–8.8 months) (Fig. 3a). Meanwhile, the median OS was 24.3 months (95% CI 14.4–41.1 months) (Fig. 3b).

**Table 2:** Responses in the intention-to-treat population

	**Independent centralized review**
ORR (95%CI)	37.8(26.8–49.9)
CR	3(4.1)
VGPR	5(6.8)
PR	20(27.0)
SD	32(43.2)
PD	5(6.8)
Not evaluable	9(12.1)^a^
TTR, months	1.84(0.95–2.35)
Median (95%CI) DOR	8.8(6.0–14.7)

*CR* complete response, *DCR* disease control rate, *DOR* duration of remission, *ORR* overall response rate, *OS* overall survival, *PD* progressive disease, *PFS* progression-free survival, *PR* partial response, *SD* stable disease, *TTR* time to treatment response, *VGPR* very good PR

^a^The patients were not evaluable as they failed to complete one cycle of treatment

### Safety

The incidence of grade 3 and above AEs was 89.2%. As shown in Table 3, the incidence of SAEs was 52.7%. Death occurred in 8.1% (6/74) patients, mostly during hospitalization. Four deaths were treatment-related lung infection, respiratory failure, or anemia, and 2 deaths were not treatment related, due to cardiac failure in one case and PD in the other. The most common grade 3 and 4 treatment-emergent AEs (TEAEs) were neutropenia (63.5%), leukopenia (37.8%), thrombocytopenia (28.4%), and anemia (31.1%). Pulmonary infections (27.0%) were the most frequent grade 3 and 4 nonhematologic TEAEs. In addition, 70.3% patients received preventative antithrombosis therapy. No venous thromboembolism was reported. There was no previously unreported AE with pomalidomide and low-dose dexamethasone in the study population. Pomalidomide dose reductions and dose interruptions due to AEs occurred in 17 (23.0%) and 23 (31.1%) patients, respectively. Seven (9.5%) patients discontinued pomalidomide because of AEs.

**Table 3:** Grade 3/4 AEs occurring in ≥ 2% of patients^a^

	**Total**	**Grade 3**	**Grade 4**
**Hematologic TEAEs**			
Neutropenia	64(86.49)	31(41.89)	16(21.62)
Leukopenia	64(86.49)	26(35.14)	2(2.70)
Thrombocytopenia	58(78.38)	14(18.92)	7(9.46)
Anemia	50(67.57)	22(29.73)	1(1.35)
Lymphocytopenia	37(50.00)	10(13.51)	5(6.76)
**Non-hematologic TEAEs**			
Hypoalbuminemia	38(51.35)	2(2.70)	0
Hyperglycemia	34(45.95)	4(5.41)	0
Proteinuria	32(43.24)	3(4.05)	0
Upper respiratory tract infection	28(37.84)	2(2.70)	0
Pyrexia	21(28.38)	3(4.05)	0
Pulmonary infection	21(28.38)	19(25.68)	1(1.35)
Hyperuricemia	18(24.32)	1(1.35)	2(2.70)
Reduced creatinine clearance (< 60 mL/min/1.73 m^2^)	17(22.97)	2(2.70)	0
Hypophosphatemia	10(13.51)	3(4.05)	0

^a^Unless otherwise noted, all data are n(%)

## Discussion

Two pivotal clinical studies MM-002 and MM-003 have established the efficacy of the original pomalidomide in combination with low-dose dexamethasone in patients with RRMM who had received at least 2 prior therapies including bortezomib and lenalidomide [15–17]. However, no such clinical data has been established in Chinese RRMM patients. The current single-arm study has demonstrated that generic pomalidomide in combination with low-dose dexamethasone is also effective and safe for Chinese patients who had received at least two previous treatments, including bortezomib and lenalidomide, offering a much-needed treatment option for this group of MM patients in China, especially those who have become resistant to lenalidomide.

The ORR of our current study (37.8%) is comparable to that of MM-002 (33%) and MM-003 (31%) while the median PFS of our patients (5.7 months) is numerically longer than that of MM-002 (4.2 months) and MM-003 (4.0 months), which could be due to the low proportion of our patients receiving autologous stem cell transplantation (10.81%) (MM-002: 74% and MM-003: 60%) and a smaller number of prior lines of therapy. In addition, the median OS of our patients reached slightly above 2 years (24.3 months), almost doubling that of MM-003 (12.7 months). Apart from the low percentage of patients who had received autologous stem cell transplantation and fewer prior lines of therapy, novel agents such as ixazomib and daratumumab, a human monoclonal antibody that targets CD38, have become available in China, which help improving the survival of patients in this trial.

Approximately one-third of our patients had renal insufficiency and they achieved an ORR of 34.8%, which is comparable to that of the whole population in this trial, suggesting that pomalidomide could also benefit RRMM patients with renal insufficiency [18]. In patients with renal insufficiency, the plasma concentration and half-life of lenalidomide are significantly higher, and lower doses of lenalidomide are used. Pomalidomide is metabolized predominantly in the liver and only 2% is excreted unchanged in the urine [19]. It is of note that a pooled analysis of three clinical trials showed no significant difference in ORR in patients with moderate renal insufficiency *versus* those without (30.4% *vs.* 33.8%) [20]. This characteristics of pomalidomide is also reflected in Chinese patients.

The ORR of MM patients with high-risk cytogenetics in our study was 36.0%, which is similar to that for the high-risk subgroup in the MM-003 trial. These findings suggest that pomalidomide could benefit MM patients and at least partially improve the adverse impact of high-risk cytogenetics in MM patients.

Our study confirmed the tolerable safety profile of pomalidomide in combination with low-dose dexamethasone, which is overall consistent with that of Caucasian MM patients, and no previously unreported AE was observed. Neutropenia (63.5%) remained the most common grade 3 and 4 TEAE and the incidence is higher than that of the MM-003 trial (48%), suggesting that greater caution should be exercised in Chinese patients. Though hematologic toxicities remained the main AEs in our patients, they were manageable by pomalidomide dose adjustment. The percentage of patients who required pomalidomide dose reduction in our study (23%) is comparable to that of the MM-003 trial (27%) [21]. The incidence of grade 3 and above pneumonia (27.0%) is higher than that of the MM-003. In addition, treatment-related death occurred in 4 (5.4%) cases, which is similar to 4% in Caucasians in MM-003 trial and comparable to 8.3% in Asian MM patients in the AMN-001 trial [21]. Besides, thalidomide, lenalidomide, and pomalidomide have been reported to increase the risk of venous thromboembolism in MM patients [22]. However, no venous thromboembolism was reported in this trial though only 70% of the patients received antithrombotic therapy. NCCN guidelines recommend that antithrombotic therapy be given to MM patients taking lenalidomide or other similar agents. However, several studies from Chinese MM patients had demonstrated that there was a low rate of venous thromboembolism for MM patients, although receiving lenalidomide or thalidomide. [23–25]. So in the current trial, antithrombotic therapy was not mandatory and was provided at the discretion of the attending physicians during the trial.

Our study has several limitations. First, this study is a single arm study, which may result in a bias in clinical assessment. Setting ORR as the primary end point is another limitation. PFS and OS should be confirmed by randomized controlled trials with a larger population. Nevertheless, it is necessary to timely verify the efficacy and safety of pomalidomide in Chinese RRMM patients, given that third-generation immunomodulatory drugs are being widely used in many countries in the world, including other countries in Asia. These findings would accelerate the development of third-generation immunomodulatory drugs in China, bringing in earlier access by Chinese RRMM patients to an alternative safe and effective drug. Though generic pomalidomide plus low-dose dexamethasone doublet treatment has been approved by China NMPA based on this trial, triplet or quadruplet regimen is currently the main stay of therapy. Pomalidomide, as a third-generation immunomodulatory drug showing a greater clinical benefit ratio, may offer a cost-effective alternative to the main triplet or quadruplet regimen, and may also act in concert with drugs of different mechanisms. In this regard, the OPTIMISMM study has also demonstrated that pomalidomide in combination with bortezomib yields greater clinical benefits than the doublet regimen with pomalidomide. The AMN-001 trial also showed that the pomalidomide-cyclophosphamide combination conferred greater benefits on Asian patients. Daratumumab-pomalidomide regimen is also being explored as a treatment for MM patients as in the APOLLO trial that compared daratumumab plus pomalidomide and dexamethasone with pomalidomide and dexamethasone, showing markedly improved PFS (median 12.4 months *vs*. 6·9 months; HR 0·63 [95% CI 0·47–0·85], *P* = 0·002) [26]. Generic preparations of original formulations are commonly used due to their low cost and bioequivalence. The efficacy and safety of generic pomalidomide in other scenarios of MM treatment need to be examined in the future. Therefore, generic pomalidomide may be further explored in novel combination regimens with other agents for Chinese RRMM patients or in MM patients who have failed lenalidomide (NCT04989140 and others).

## Conclusion

The generic pomalidomide in combination with low-dose dexamethasone offers an efficacious and safe treatment in Chinese RRMM patients. With the increasing use of VRd induction therapy in China, the number of Chinese RRMM patients refractory to lenalidomide will inevitably rise. Therefore, the introduction of generic pomalidomide into China could offer an appealing low-cost, effective and tolerable treatment option for this group who currently lack effective therapies.

## Supplementary Information

**Additional file 1.** Bioequivalence of two pomalidomidecapsules in healthy Chinese male subjects.

**Additional file 2.** List of participating institutions.

**Additional file 3.** Supplementary Methods.

## Data Availability

The data that support the findings of this study are available from Chia Tai Tianqing Pharmaceutical Group Co. Ltd., but restrictions apply to the availability of these data, which were used under license for the current study, and so are not publicly available. Data are however available from the authors upon reasonable request and with permission of Chia Tai Tianqing Pharmaceutical Group Co. Ltd. (for inquiries, yadong.miu@cttq.com).

## Abbreviations

MM: Multiple myeloma
VRd: Bortezomib, lenalidomide, and dexamethasone
RRMM: Relapsed/refractory
NMPA: National Medical Products Administration
ECG: Electrocardiography
IRAC: Independent Review Advisory Committee
ITT: Intention-to-treat
DOR: Duration of response
PFS: Progression-free survival
OS: Overall survival
SAEs: Severe AEs
FAS: Full analysis set
PPS: Per protocol set
CI: Confidence interval
