# Supplementary material for: Efficacy and safety of pomalidomide and low-dose dexamethasone in Chinese patients with relapsed or refractory multiple myeloma: a multicenter, prospective, single-arm, phase 2 trial
Source: BMC Cancer. 2022 Jul 1;22:722. doi: 10.1186/s12885-022-09802-y (PMC9250185; doi:10.1186/s12885-022-09802-y)
Supplement: Supplementary file 1 — Additional file 1. Bioequivalence of two pomalidomidecapsules in healthy Chinese male subjects. [file 12885_2022_9802_MOESM1_ESM.docx]

**Additional file 1**

**Bioequivalence of two pomalidomide capsules in healthy Chinese male subjects**

LIU Zheng-zhi^1^, XU zhong-nan^2^, GAO zhen-yue^2^, REN Qing^1^, CHANG Tian-ying^1^, XUE jin-ling^2^, YANG Hai-miao^1^

(1. Changchun University of Chinese Medicine Affiliated Hospital, Institute of Phase I Clinical Trial, Changchun 130012, 2. Chai Tai Tianqing Pharmaceutical Group co., Ltd)

**[Summary]**

**OBJECTIVE:** To evaluate the pharmacokinetic characteristics of pomalidomide capsules in healthy Chinese male subjects, and to evaluate the bioequivalence of the two preparations at 1mg and 4mg doses, respectively.

**METHODS:** At 1 mg and 4 mg doses, fasting and fed state bioequivalence tests were conducted.24 healthy male subjects were recruited for each dose group, fasting state and fed state. The test reagent and pomalidomide capsules (Imnovid^®^) were taken orally. A randomized, open, two-cycle, two-crossing fasting state and self-controlled fed state test method was adopted. The plasma drug concentrations at 17 different time points within 48 h were determined by liquid chromatography-tandem mass spectrometry (lc-ms). The main pharmacokinetic parameters Cmax, AUC_0-t_ and AUC_0-∞_ were calculated to evaluate the bioequivalence.

**RESULTS:** In the 1 mg dose group, the geometric mean ratios of main pharmacokinetic parameters Cmax, AUC_0-t_ and AUC_0-∞_ were 95.06%, 94.24% and 94.64%, respectively, and the 90% confidence intervals were 87.91%-102.80%, 89.73%-98.99% and 90.29-99.19% under fasting state, respectively, within the range of 80.00% to 125.00%, meeting the criteria for bioequivalence. The ratios of geometrical mean values of main pharmacokinetic parameters Cmax, AUC_0-t_ and AUC_0-∞_ in the 1mg dose group were 94.98%, 96.27% and 96.19% in the fed state, respectively, and the confidence intervals of 90% were 88.34-102.11%, 89.84-103.17% and 89.82-103.02%, respectively, which ranged from 80.00% to 125.00%, meeting the criteria of bioequivalence. In the 4mg dose group, the geometric mean ratios of main pharmacokinetic parameters Cmax, AUC_0-t_ and AUC_0-∞_ were 103.62%, 106.53%, 107.00%, and 90% confidence intervals were 95.79-112.09%, 102.85-110.35%, and 103.40-110.73%, respectively, after oral administration of test preparations and reference preparations under fasting state, which met the criteria of bioequivalence. In the 4mg dose group, the geometric mean ratios of main pharmacokinetic parameters Cmax, AUC_0-t_ and AUC_0-∞_ were 109.68%, 103.16% and 102.78%, respectively, and the 90% confidence intervals were 103.37-116.36%, 100.69-105.69% and 100.34-105.29% in the fed state, respectively, which were within the range of 80.00% to 125.00%, meeting the criteria for bioequivalence.

**Conclusion:** Both 1 mg and 4 mg doses of pomalidomide showed bioequivalence in both the fasting and fed state.

**[Key words]** Pomalidomide capsules; multiple myeloma; LC-MS/MS; bioequivalence
