# Supplementary material for: Efficacy and safety of pomalidomide and low-dose dexamethasone in Chinese patients with relapsed or refractory multiple myeloma: a multicenter, prospective, single-arm, phase 2 trial
Source: BMC Cancer. 2022 Jul 1;22:722. doi: 10.1186/s12885-022-09802-y (PMC9250185; doi:10.1186/s12885-022-09802-y)
Supplement: Supplementary file 2 — Additional file 2. List of participating institutions. [file 12885_2022_9802_MOESM2_ESM.docx]

**Additional file 2 List of participating institutions**

Changzheng Hospital, Shanghai, China.

Tianjin Medical University Cancer Institute and Hospital, Tianjin, China.

The Affiliated Hospital of Qingdao University, Qingdao, Shandong Province, China.

West China Hospital, Sichuan University, China.

Affiliated Cancer Hospital of Zhengzhou University and Henan Cancer Hospital, Zhengzhou, China.

Shengjing Hospital of China Medical University, Shenyang, Liaoning, China.

The 940th Hospital of Joint Logistics Support force of Chinese People’s Liberation Army, Lanzhou, China.

Peking University People's Hospital and Peking University Institute of Hematology, Beijing, China.
