# Supplementary material for: Efficacy and safety of pomalidomide and low-dose dexamethasone in Chinese patients with relapsed or refractory multiple myeloma: a multicenter, prospective, single-arm, phase 2 trial
Source: BMC Cancer. 2022 Jul 1;22:722. doi: 10.1186/s12885-022-09802-y (PMC9250185; doi:10.1186/s12885-022-09802-y)
Supplement: Supplementary file 3 — Additional file 3. Supplementary Methods. [file 12885_2022_9802_MOESM3_ESM.docx]

**Supplementary Methods**

**Eligibility criteria**

We included 1) patients who had measurable disease with at least one of the following: a) serum M protein level was ≥ 0.5 g/dL, b) 24-h urinary M protein level ≥ 200 mg, or c) the involved FLC level ≥ 10 mg/dL provided serum FLC ratio is abnormal; 2) an ECOG performance score of 0 to 2; 3) life expectancy ≥ 3 months; 4) a washout period of ≥ 14 days from the most recent therapy excluding dexamethasone; 5) patients were able to use antithrombotic agents such as low molecular weight heparin or aspirin. Other main inclusion criteria were 1) an absolute neutrophil count (ANC) ≥1.0×10^9^/L including under G-CSF support and platelet count ≥ 50×10^9^/L; 2) total bilirubin ≤ 2.0 mg/dL, aspartate aminotransferase (ALT) and aspartate aminotransferase (AST) ≤ 3.0 times the upper limit of normal, 3) serum creatinine ≤3.0 mg/dL or calculated creatinine clearance ≥ 30 mL/min, and 4) platelet count ≥ 30 ×10^9^/L for whom ≥50% of bone marrow nucleated cells were plasma cells. Patients were excluded if they were unable to tolerate thalidomide, lenalidomide, pomalidomide or other drugs of the same class at the discretion of the investigators, or they had known allergy to pomalidomide or similar immune modulators and dexamethasone, or their excipients. Non-secretory or oligo-secretory MM (involved light chain < 100 mg/L) patients were also excluded. We further excluded patients with active *de novo* thrombosis or who declined anti-thrombosis therapy. Other exclusion criteria were 1) concurrent or previous malignancy excluding basal cell carcinoma, cutaneous squamous cell carcinoma, cervical carcinoma *in situ*, breast carcinoma *in situ*, TNM T1a or T1b prostate cancer or prostate cancer under treatment; 2) receipt of anti-tumor therapy including major surgery within 4 weeks of the study; 3) requiring long term immunosuppressants or steroid drugs; 4) known active hepatitis B (HBV DNA≥1×10^3^ copies/mL) or C (HCV) or positive HIV serology; 5) concurrent severe infectious diseases; 6) contemplating pregnancy or failure to take reliable contraceptive measures; 7) NYHA class 2 or above, myocardial infarction within the preceding year, or uncontrolled angina pectoris including variant angina pectoris; 8) central nervous system (CNS) disease requiring treatment and peripheral neuropathy grade ≥3; 9) receipt of allogeneic stem cell transplantation within the preceding year, or active G*v*HD) or G*v*HD requiring immunosuppressant therapy if receipt of allogeneic stem cell transplantation for more than 1 year; 10) participation in other clinical trials within the preceding month. Pregnant or lactating women were also excluded.
